# When Can a Computed Pt(II) Substitution Rate Be Trusted? A DFT Framework with Four Reliability Diagnostics Applied to Cisplatin

**DOI:** 10.3390/ijms27219887

**Published:** 2026-11-04

**Authors:** Maciej Spiegel, Marcin Mączyński

**Affiliations:** Department of Organic Chemistry and Pharmaceutical Technology, Faculty of Pharmacy, Wroclaw Medical University, Borowska 211 A, 50-556 Wroclaw, Poland

**Keywords:** cisplatin, platinum drugs, DFT, transition-state theory, kinetics, aquation, thiolation, precomplex, diffusion limit, selectivity, guanine substitution

## Abstract

This study presents a DFT protocol for the substitution kinetics of Pt(II) complexes, applied to cisplatin as a case study. We aim to demonstrate that a computed barrier that agrees with experiment does not, by itself, validate the kinetic model, and that further diagnostics are required to assess the reliability of the results. M06-2X/x2c-TZVPall calculations with implicit solvation, explicit treatment of one coordinating water molecule and a set of physical corrections were used to study the aquation, thiolation (with a capped cysteine-thiolate model) and guanine substitution (by 9-methylguanine) pathways. The calculated precomplex-to-transition-state barrier for the first aquation is 2.0 kcal⋅mol^−1^ below the Eyring barrier inferred from experiment; with the symmetry factor σ = 2 applied, the calculated observed rate constant is 56-fold higher than the experimental value. The formal low-concentration second-order coefficient for thiolation exceeds the reported glutathione rate coefficient by 1.5–1.8 log units, whereas omission of the physical corrections shifts the calculated rate predictions by about 5–8 log units. A direct selectivity descriptor (*S*_direct_) is introduced and shown to be governed by the precomplex term rather than by the activation step, making it vulnerable to precomplex overstabilization by the implicit solvent. Four diagnostics are proposed that should accompany any computed rate constant or selectivity: the sign of the precomplex free energy, the magnitude of *K*_pre_, the proximity to the diffusion limit, and the decomposition of selectivity into precomplex and activation contributions. These provide a systematic basis for assessing the reliability of computed predictions.

## 1. Introduction

Although cisplatin was first synthesized by Michele Peyrone in 1844, its biological activity remained unrecognized until 1965, when Rosenberg and co-workers serendipitously observed inhibition of cell division in *E. coli* [1]. The mechanism underlying the anticancer activity of cisplatin was elucidated only gradually—early studies demonstrated that the aquated form interacts with DNA, particularly at the N^7^ position of guanine, generating 1,2-intrastrand cross-links, and these DNA lesions were subsequently shown to induce cell-cycle arrest and, ultimately, apoptotic cell death via p53 signaling and activation of the caspase cascade [2].

Over time, this mechanistic explanation has evolved considerably: aquated platinum species were found to interact not only with DNA but also with other strong intracellular nucleophiles, particularly sulfur-containing biomolecules—glutathione, metallothioneins, and other cysteine-rich proteins. Thioether donors also constitute a distinct class of sulfur nucleophiles: monodentate *S*-bound methionine is able to coordinate reversibly to platinum. In addition, methionine-rich motifs of the copper transporter CTR1 have been implicated in direct interactions with cisplatin [3] and have been shown to form mixed-ligand platinum–guanine adducts [4]. The literature further indicates that Pt(II) complexes can react directly with sulfur nucleophiles without prior aquation—otherwise a prerequisite for their activation—thereby providing an activation-independent pathway for platinum sequestration and diminishing the fraction available for DNA platination [5,6]. Platinum–glutathione adducts have also been shown to undergo subsequent reactions with 5′-GMP, demonstrating that sulfur-bound platinum species can participate in downstream guanine-directed transformations [7,8,9]. These platinum–sulfur adducts have been repeatedly implicated in platinum-drug detoxification and in resistance mechanisms, and the resulting resistance remains a major obstacle in clinical oncology. The reaction pathways of platinum complexes depend strongly on the chemical environment; specifically, the partitioning of cisplatin between neutral and cationic aquated species depends on both pH and chloride concentration [10]. Heudi et al. [11] found that *L*-methionine reacts more rapidly with the mono-aquated species than with non-aquated cisplatin itself, demonstrating that the aquation state of the platinum center substantially affects its reactivity towards sulfur nucleophiles. More broadly, the reaction pathway is known to depend on the reaction medium, proceeding through mono-aquated species in water, phosphato derivatives in phosphate buffer, or direct reaction of cisplatin with *L*-methionine in the presence of chloride ions [12]. Thus, the kinetically preferred initial interaction and the ultimate platinum target may represent distinct steps within the same reaction network.

At the molecular scale, computational chemistry has long been addressing Pt(II) complexes, establishing the importance of explicit solvation and the pitfalls in interpreting hydrolysis barriers from implicit-solvent calculations [13,14], as well as the binding of Pt(II) to purine bases, providing a framework for understanding the competition between guanine and adenine [15,16], among other topics. Nevertheless, studies to date have predominantly focused on the reaction energies and activation barriers of individual steps, whereas the influence of encounter-complex formation, standard-state effects, and diffusion on the experimentally observed rate remains comparatively underexplored—despite both aquation and the subsequent substitution proceeding under kinetic control [16,17,18,19]. It is acknowledged that theoretical models cannot recapitulate the behavior of a drug within the complex environment of a cancer cell—accordingly, comparison with experimentally determined rate constants and barriers in well-defined media remains the most direct means of assessing a computational protocol: discrepancies expose methodological choices that neglect features that are readily overlooked, whereas agreement, as demonstrated below, does not in itself establish the validity of the model.

The present study employs cisplatin as a proof of concept to demonstrate how treating or omitting each micro-step explicitly affects the observed reactivity and may lead to false conclusions. A barrier that is readily computed and agrees with experiment is not, by itself, evidence of a physically correct model, because it can be reached through cancellation of errors, particularly when the corrections that account for the physics of association are omitted. To this end, we computed the aquation of cisplatin and its reactions with a thiolate and a guanine model, quantified the impact of each correction and protocol setting, and proposed four diagnostics for assessing the reliability of computed Pt(II) substitution rates.

## 2. Results and Discussion

### 2.1. Reaction Profiles of Cisplatin with Water, Thiolate and Guanine

**Figure 1:**
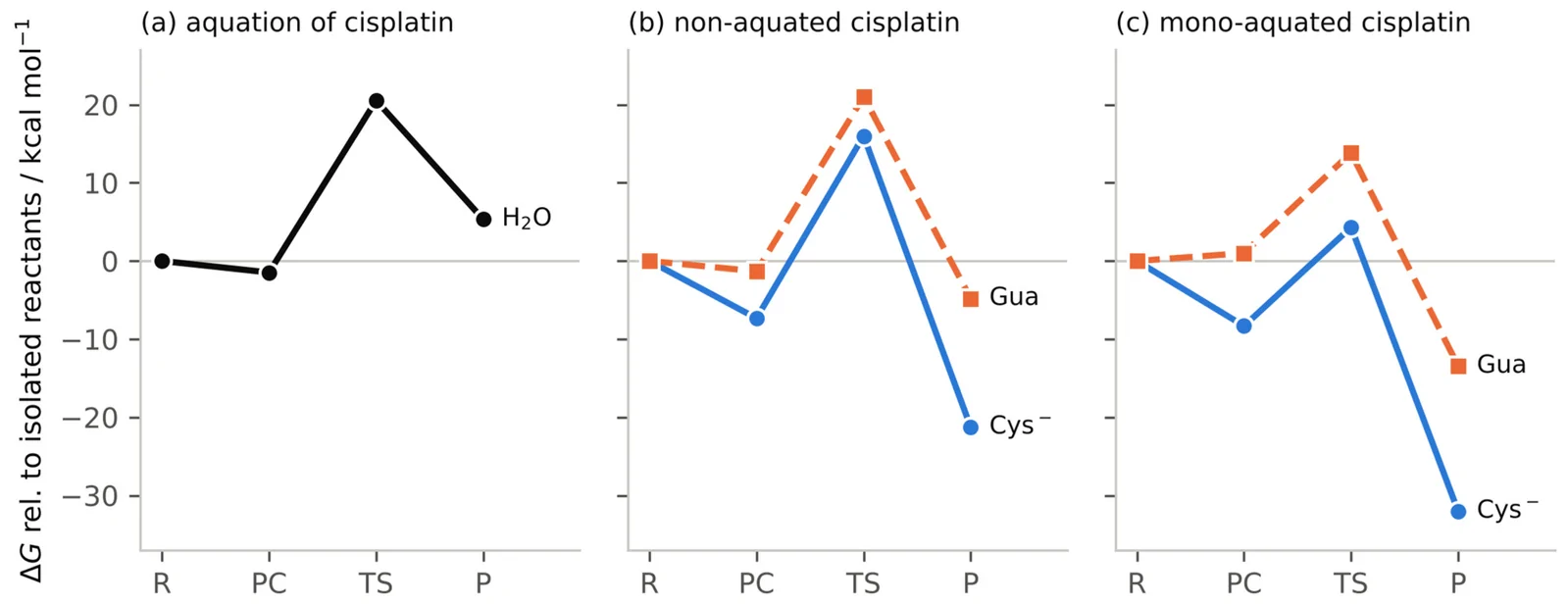
Calculated free-energy profiles (kcal⋅mol^−1^, relative to the isolated reactants, 298.15 K) for (**a**) the first aquation of cisplatin, and the attack of thiolate (blue, solid) and 9-methylguanine (orange, dashed) on (**b**) non-aquated and (**c**) mono-aquated cisplatin. R, reactants; PC, precomplex; TS, transition state; P, postcomplex.

Figure 1 and Table 1 present the calculated profiles for the aquation of cisplatin and for attack by thiolate and guanine on its non-aquated and mono-aquated forms, obtained following the methodology in Section 3. Hereafter, *K*_pre_ denotes the concentration-based equilibrium constant for precomplex formation from the isolated reactants, and *S*_direct_ is the direct selectivity descriptor, defined as the base-10 logarithm of the ratio of the apparent second-order rate coefficients for thiolate and guanine.

The calculated free-energy barrier from the precomplex to the transition state (ΔG^‡^_pre→TS_) for the first aquation of cisplatin is 22.0 kcal⋅mol^−1^. Given that the experimental rate constant (*k*_exp_) at 298.15 K equals 1.62 × 10^−5^ s^−1^ [17]—which corresponds to an activation free energy of 24.0 kcal⋅mol^−1^—the calculated barrier is only 2.0 kcal⋅mol^−1^ lower than the experimental one. This *k*_exp_ value is only one of many—for instance, an HPLC-ICP-MS speciation study reported first-order aquation rate constants of 1.68–2.06 × 10^−5^ s^−1^ across solutions with different chloride concentrations, including [Cl^−^] = 0 M [20]. Because the experimental solution conditions differ, these values are not treated as interchangeable benchmarks, but they provide an independent reference for the magnitude of the aquation rate. The rate constant also includes an additional symmetry factor *σ* = 2 for non-aquated cisplatin, since the two chloride ligands are equivalent; therefore, the corresponding calculated observed rate constant (*k*_obs_) is estimated at 9.06 × 10^−4^ s^−1^ and exceeds the experimental value by a factor of about 56 (1.75 log units).

This first comparison alone yields three observations: firstly, the 2.0 kcal⋅mol^−1^ difference between the calculated and Eyring-derived barriers appears modest on the energy scale, yet together with the symmetry factor σ = 2 it produces a 56-fold rate discrepancy, equivalent to an effective free-energy difference of approximately 2.4 kcal⋅mol^−1^; secondly, the symmetry factor σ underscores why the statistical prefactor matters: omitting σ = 2 counts the two equivalent chloride ligands as distinguishable and therefore doubles the calculated rate; and thirdly, the barrier obtained here lies within the range of previously reported DFT values for cisplatin aquation, which vary substantially with the functional, basis set and solvation model [13,14,16]. The last point is particularly important because typical theoretical calculations omit the physical corrections, so agreement with experiment alone does not establish that the chosen level of theory is physically correct.

The calculated thiolation and guanine substitution pathways differ substantially in their precomplex stabilization. The results suggest that in the non-aquated state, the thiolate precomplex is stabilized by 7.3 kcal⋅mol^−1^ (*K*_pre_ = 2.4 × 10^5^ M^−1^) relative to the separated reactants, compared with 1.3 kcal⋅mol^−1^ for the guanine precomplex (Δ*G* = –1.3 kcal⋅mol^−1^, *K*_pre_ = 9.2 M^−1^). Despite this, the barrier from the precomplex is only slightly higher for the thiolate (23.3 vs 22.3 kcal⋅mol^−1^). The precomplex term therefore favors the thiolate by 6.0 kcal⋅mol^−1^, whereas the activation term favors guanine by 1.0 kcal⋅mol^−1^, resulting in a net preference toward the thiolate (*S*_direct_ = 3.7) that is carried by the encounter complex rather than by the intrinsic activation step.

Upon aquation, the calculated preference for thiolate substitution increases further: in the mono-aquated state, the barrier from the precomplex drops from 23.3 to 12.6 kcal⋅mol^−1^, the precomplex becomes more stable by 1.0 kcal⋅mol^−1^ relative to the non-aquated state (*K*_pre_ = 1.3 × 10^6^ M^−1^), and the overall barrier falls to 4.3 kcal⋅mol^−1^. The calculated *k*_app_ approaches the diffusion limit: *k*_app_ = 3.13 × 10^9^ M^−1^⋅s^−1^ compared with a diffusion-limited value of 1.20 × 10^10^ M^−1^⋅s^−1^ for the oppositely charged reactants, whose electrostatic attraction raises the diffusion-controlled rate by a factor of about 1.6. The resulting complex is strongly stabilized (Δ*G*_(post−isol)_ = −32.1 kcal⋅mol^−1^), consistent with essentially irreversible Pt–S bond formation. For guanine, the barrier is 12.8 kcal⋅mol^−1^ (only 0.2 kcal⋅mol^−1^ higher than that of the thiolate), but the precomplex remains destabilized relative to the separated reactants (Δ*G*_(pre−isol)_ = 1.0 kcal⋅mol^−1^, *K*_pre_ = 0.18 M^−1^), and the guanine postcomplex is far less stabilized (Δ*G*_(post−isol)_ = −13.5 kcal⋅mol^−1^). The overall barrier therefore rises to 13.8 kcal⋅mol^−1^, i.e., 9.5 kcal⋅mol^−1^ above the thiolate value. This contrast, driven almost entirely by the encounter complex rather than by the activation step, raises *S*_direct_ to 6.8.

Experimental rate constants for thiolation of non-aquated cisplatin are 2.7 × 10^−2^ M^−1^ s^−1^ [21] and 1.3 × 10^−2^ M^−1^ s^−1^ [22]; they serve here as benchmarks. The calculated formal low-concentration second-order coefficient for thiolation by the glutathione-relevant thiolate model at pH 7.4 gives *k*_app_ × *f*_active_ ≈ 0.87 M^−1^ s^−1^, which exceeds the two reported coefficients by 1.5 and 1.8 log units, respectively. Both experiments were, however, carried out at 310 K, whereas the calculations refer to 298.15 K, so a temperature correction is required. A simple Eyring estimate—taking Δ*E*_el+ZPE(isol→TS)_ ≈ 3.9 kcal⋅mol^−1^ as the dominant contribution to the activation enthalpy, with ±3 kcal⋅mol^−1^ permitted for the neglected thermal enthalpy—yields *k*_(310 K)_/*k*_(298 K)_ ≈ 1.1–1.6. A like-for-like comparison would therefore increase the overestimation by no more than approximately 0.2 log units, confirming that the discrepancy cannot be ascribed to the temperature difference. This estimate treats *f*_active_ as temperature-independent; the temperature dependence of the thiol p*K*_a_ and of the buffer pH is not included. The present calculations do not isolate the contributions of the exchange–correlation functional, the implicit-solvation model and the simplified nucleophile model to the remaining discrepancy. The excessive stabilization of the calculated thiolate precomplex relative to the Fuoss contact-pair reference makes the precomplex free energy a plausible contributor to the discrepancy, but the present data do not establish whether it arises from solvation effects, genuine specific interactions, limitations of the functional, or a combination of these factors.

An additional limitation concerns the acid–base speciation of the mono-aquated complex. The acid dissociation constant reported for *cis*-[PtCl(NH_3_)_2_(H_2_O)]^+^ is p*K*_a_ = 6.56 at 37 °C [23]. Using this value as an illustrative Henderson–Hasselbalch estimate, only approximately 13% of the complex would remain in the mono-aquated form at pH 7.4, with the remainder predominantly deprotonated to the monohydroxo form. The exact fraction may differ under the model kinetic conditions because the chloride concentration and ionic environment are not identical to those under which the p*K*_a_ was determined. The present calculations include the mono-aquated form but do not include its deprotonation to the monohydroxo species. The experimental glutathione rate used here was measured at approximately pH 7.4 and reported as a second-order rate coefficient, not as a rate specific to the mono-aquated form. This omission limits the interpretation of the mono-aquated pathways under physiological pH conditions, but it does not directly alter the calculated pathway for attack on non-aquated cisplatin, which was modeled as a separate reaction.

Agreement between a calculated barrier and experiment does not, by itself, validate the underlying model—such agreement can arise from a fortuitous cancellation of errors, with some contributions overestimated and others underestimated [24,25,26,27,28,29]. The treatment of the entropic penalty of association and the number of explicit solvent molecules used for charged solutes are among the choices that can compensate for one another, yielding apparently correct outcomes. The magnitude of the corrections discussed below illustrates how readily such compensation can occur.

### 2.2. Sensitivity of the Results to Protocol Settings

The protocol presented above treats each step of the reaction as a separate choice. Three categories of protocol settings were examined to determine whether they shift the calculated rate constants below or above experimental values: these include physical corrections that account for the association step, sensitivity tests that identify where the model fails, and variations in the experimental conditions (pH) and in the literature-derived parameters (p*K*_a_). This section shows how each of these choices affects the computed rate constants and selectivity, and Table 2 summarizes the corresponding results.

Removing all three physical corrections lowers the calculated aquation rate by 5.3 log units and the calculated thiolation rate by 8.1 log units, with the Benson–Okuno correction alone accounting for 0.9 and 3.6, respectively. The pronounced disparity in the sensitivity of the two rates to precomplex free energy—aquation varying by less than 0.03 log units vs 1.5 log units for thiolation upon a 2 kcal⋅mol^−1^ shift—stems from the distinct kinetic regimes in which the respective rate constants operate. Under aquation conditions, the precomplex is saturated (*K*_pre_[H_2_O] ≈ 670), so that the observed rate constant equals σ*k*_TST_ and is independent of *K*_pre_. Although the corrections lower *K*_pre_ by 8.1 log units, 2.8 of these log units, corresponding to log(*K*_pre_[H_2_O]) ≈ 2.8, merely reduce the degree of saturation, leaving only the residual 5.3 log units to influence the rate. In thiolation, by contrast, the experimentally compared quantity is the second-order constant *k*_Cys_ = *k*_app_ × *f*_active_, which is proportional to *K*_pre_; the corrections therefore enter it in full (8.1 log units), and any error in the precomplex free energy propagates directly into the rate. The baseline value *K*_pre_ × [Nu] × *f*_active_ ≈ 9 at 1 mM total thiol indicates that the model is already in the precomplex-saturation regime at this concentration. Accordingly, *k*_Cys_ = *k_app_ × f*_active_ is the formal low-concentration second-order limit, not the model’s apparent rate coefficient at 1 mM total thiol. The finite-concentration value must instead be evaluated. Consequently, the comparison in Table 2 is a comparison of formal second-order coefficients rather than a concentration-matched validation of the observed pseudo-first-order rate at millimolar glutathione concentrations.

The diffusion-related settings (Collins–Kimball, Debye factor, and symmetry factor) change the rate constants by at most 0.3 log units and *S*_direct_ by at most 0.14 log units. A separate test replaces Δ*G* with Δ*E*_el+ZPE_—a practice still found in the literature for systems where entropic contributions are considered unreliable because of quasi-RRHO or low-frequency errors, or because a second solvation sphere is not modeled—which raises the aquation rate by about 1.0 log unit, lowers the thiolation rate by 0.2 log units, and lowers *S*_direct_ for the mono-aquated form by 0.7. Taken together, these tests show that the absolute rates are far more sensitive to the physical corrections than to the diffusion-related settings or to the use of Δ*E* instead of Δ*G*. The two rates differ, however, in their sensitivity to the precomplex free energy: for thiolation, a 2.0 kcal⋅mol^−1^ shift changes the rate by 1.5 log units, whereas for aquation the effect is negligible (0.03 log units) because the precomplex is saturated.

Changing the number of explicit water molecules by one alters the standard-state and Benson–Okuno corrections by approximately 4.4 kcal⋅mol^−1^, and microsolvation studies of cisplatin hydrolysis, such as the study by de Cózar et al. [30], demonstrate that the computed barriers are sensitive to the number of explicit waters. The present protocol therefore employs one coordinating water in every precomplex, transition state and postcomplex, so that the number of explicit waters remains identical for all species compared and the entropy of association is accounted for through the corrections. Additional explicit waters could alter the calculated precomplex energetics by changing first-shell solvation and specific interactions, but the direction and magnitude of this effect cannot be inferred from the present calculations. Inclusion of additional explicit waters therefore cannot be assumed to remove the excessive precomplex stabilization, and models with additional explicit water molecules were not tested here.

The magnitude of the calculated precomplex stabilization can be assessed against the association constant *K*_F_ of a non-specific contact ion pair. Table 3 provides such data: for the thiolate, *K*_F_ = 0.86 M^−1^ (non-aquated) and 2.26 M^−1^ (mono-aquated), whereas the calculated *K*_pre_ values of 2.4 × 10^5^ M^−1^ and 1.3 × 10^6^ M^−1^ correspond to stabilizations exceeding those of a contact pair by 7.4 and 7.8 kcal⋅mol^−1^, respectively. Conversely, for guanine, the excess is only 1.5 kcal⋅mol^−1^ for the non-aquated form and is negative (−0.8 kcal⋅mol^−1^) for the mono-aquated form, in line with the weak stabilization of the former and the destabilization of the latter. The effective constant required to reproduce the experimental glutathione rate (3.5 × 10^3^–7.3 × 10^3^ M^−1^) is still 4.9–5.4 kcal⋅mol^−1^ above *K*_F_. Because *K*_F_ models only non-specific electrostatic contact, and specific interactions can either increase or decrease the actual association constant, it serves as a diagnostic reference rather than a correction.

**Figure 2:**
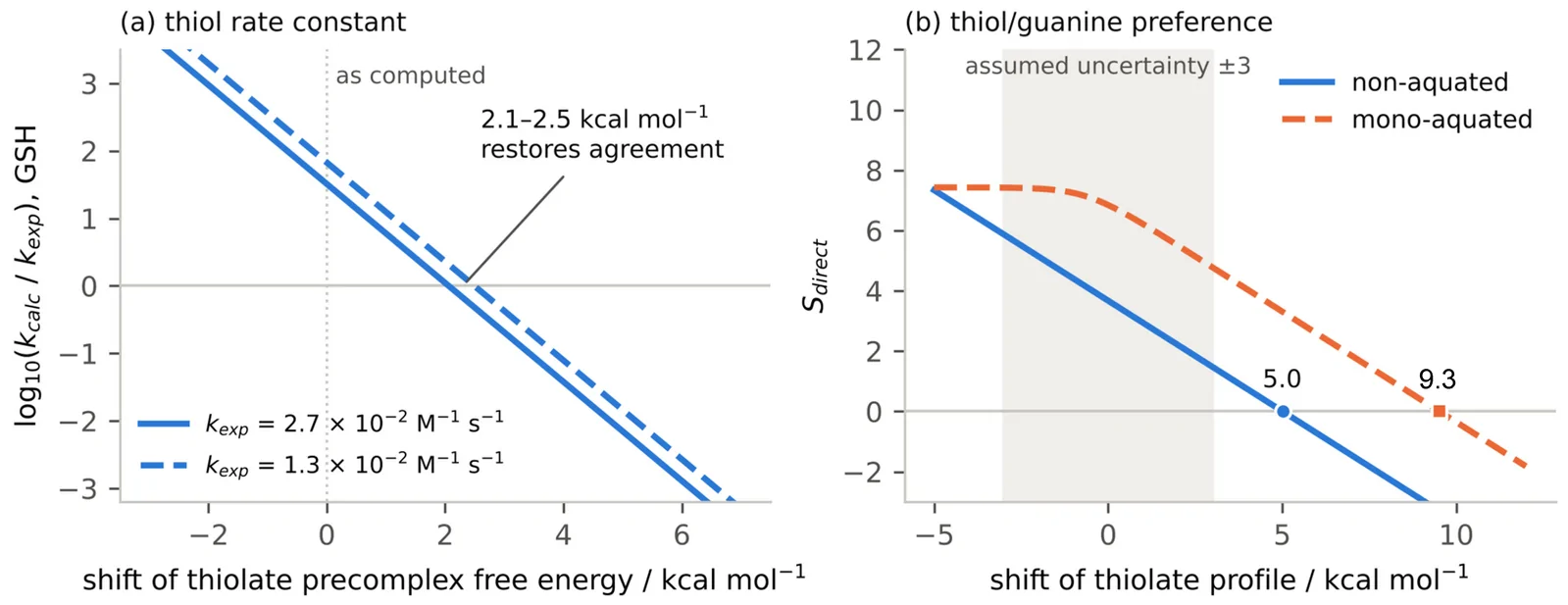
(**a**) Deviation of the calculated rate constant for the reaction of non-aquated cisplatin with glutathione from experiment, as a function of a shift in the thiolate precomplex free energy (positive = less stable). (**b**) thiolate/guanine preference *S*_direct_ as a function of a shift in the thiolate profile relative to the isolated reactants; markers give the shift at which the preference vanishes. The gray band marks the ±3 kcal⋅mol^−1^ uncertainty.

The influence of a weakened encounter complex on the predicted selectivity can be assessed directly. When *K*_pre_ is capped at 10^2^ M^−1^, *S*_direct_ decreases from 6.84 to 2.88 for the mono-aquated form and from 3.67 to 0.30 for the non-aquated form. A less restrictive cap at 10^4^ M^−1^ still lowers *S*_direct_ to 4.88 for the mono-aquated form and to 2.30 for the non-aquated form. The two capped predictions bracket the experimental glutathione rate for the non-aquated pathway (log(*k*_Cys_/*k*_exp_) = −1.86 at 10^2^ M^−1^ and 0.14 at 10^4^ M^−1^); exact agreement would require the thiolate precomplex to be 2.1–2.5 kcal⋅mol^−1^ less stable than calculated (Figure 2a). This shift falls within the ±2–3 kcal⋅mol^−1^ uncertainty considered for the calculated barriers and is consistent with a discrepancy of the magnitude expected from a single erroneous contribution.

The results indicate that the sign of the selectivity is robust to errors of this magnitude; to erase the preference for the thiolate (i.e., to make *S*_direct_ = 0), the thiolate profile would have to be less stable by 5.0 kcal⋅mol^−1^ (non-aquated) or 9.3 kcal⋅mol^−1^ (mono-aquated) relative to guanine (Figure 2b). If the two profiles carry independent errors of 2 kcal⋅mol^−1^, *S*_direct_ has a standard deviation of 2.1 log units, and the probability that the true value is negative is approximately 4% for the non-aquated form and below 0.1% for the mono-aquated form. The magnitude of *S*_direct_, however, changes by 1.5 log units per 2 kcal⋅mol^−1^. Variations in thiol pH between 7.0 and 7.8 and in thiol p*K*_a_ between 8.5 and 9.2 move *k*_Cys_ by at most 0.4 log units relative to the baseline and leave *S*_direct_ unchanged. These estimates assume that the errors in the two profiles are independent and Gaussian, and that the precomplex and barrier errors within each profile are uncorrelated.

### 2.3. Diagnostics

Despite the simplicity of the system, the results obtained for cisplatin reveal where studies on Pt(II) complexes are least reliable. To address these gaps, four diagnostics drawn from the cisplatin results are proposed as auxiliary tools that should accompany any computed rate constant or selectivity:

(i) *Sign of the precomplex free energy*. A positive Δ*G*_(pre−isol)_ indicates that precomplex formation is thermodynamically unfavorable under the chosen standard state. It does not invalidate the corresponding formal *K*_pre_, but a *K*_pre_ value below 1 M^−1^ should not be interpreted as evidence for a strongly populated, stable encounter complex. For cisplatin, this applies to the guanine attack on mono-aquated cisplatin (Δ*G*_(pre-isol)_ = 1.0 kcal⋅mol^−1^, *K*_pre_ = 0.18 M^−1^), for which the calculated association is unfavorable relative to the separated reactants.
(ii) *Magnitude of* *K*_pre_. *K*_pre_ should be compared with the Fuoss estimate *K*_F_ for a non-specific contact ion pair. Excess stabilizations of several kcal⋅mol^−1^ relative to *K*_F_ may indicate overstabilization by the implicit solvent or specific interactions that need to be justified. For cisplatin, the thiolate precomplex exceeds *K*_F_ by 7.4 (non-aquated) and 7.8 (mono-aquated) kcal⋅mol^−1^, whereas the guanine precomplex exceeds it by only 1.5 kcal⋅mol^−1^ (non-aquated) and lies 0.8 kcal⋅mol^−1^ below *K*_F_ (mono-aquated).
(iii) *Proximity to the diffusion limit*. When *k*_equiv_ approaches *k*_D_, the Collins–Kimball correction compresses the rate constant and, consequently, any selectivity descriptor derived from it. For the thiolate with mono-aquated cisplatin, *k*_equiv_ = 4.2 × 10^9^ M^−1^ s^−1^ is within a factor of 3 of the Debye-corrected *k*_D_′ = 1.2 × 10^10^ M^−1^ s^−1^. The effect is small here (without the correction, *S*_direct_ would be 7.0 instead of 6.8; see Table 2), but any further stabilization of the precomplex would be masked by the Collins–Kimball correction: the mono-aquated curve in Figure 2b flattens at *S*_direct_ ≈ 7.4.
(iv) *Decomposition of selectivity*. The selectivity should be decomposed into its precomplex and activation contributions. If the precomplex term dominates, the result rests on the part of the model that is most sensitive to protocol choices and should be reported with corresponding caution. We define the precomplex-level preference as *S*_pre_ = log_10_(*K_pre_*,_Cys_/*K_pre_*,_Gua_). In cisplatin, the precomplex term dominates in both the non-aquated (*S*_pre_ = 4.4) and the mono-aquated (*S*_pre_ = 6.8) forms; in the non-aquated form the barrier term partially offsets this preference, whereas in the mono-aquated form it slightly reinforces it. The near-equality of *S*_pre_ and *S*_direct_ (6.8) in the latter case is therefore coincidental: the small thiolate-favoring barrier term nearly cancels the Collins–Kimball compression.

These four diagnostics are formulated in general terms and are expected to be applicable beyond cisplatin, although this was not tested here. For other Pt(II) drug candidates, the same checks can be applied once the dominant reacting species, ligand-exchange pathway and relevant acid–base equilibria are specified. For example, the ligand-exchange profiles of carboplatin and oxaliplatin must be assessed for their own coordination environments rather than inferred from cisplatin. The present study therefore supports transferring the diagnostic questions, not numerical *K*_pre_, diffusion-related, or selectivity values, without system-specific calculations. Any Pt(II) substitution reaction may exhibit a precomplex free energy of the wrong sign, an overstabilized encounter complex, a rate constant compressed by the Collins–Kimball correction near the diffusion limit, or a selectivity dominated by non-covalent interactions. Different systems will trigger different diagnostics, and the systematic application of all four reduces the risk that a problematic result is overlooked.

## 3. Materials and Methods

All quantum-mechanical calculations were performed with ORCA 6.1.0 [32]. Global-minimum structures were sampled with the GFN2-xTB method [33] and the ALPB implicit water model [34] using the GOAT conformer-search module [35]. Isolated structures, precomplexes, postcomplexes and transition states were optimized with the M06-2X functional [36], the exact two-component (x2c) relativistic Hamiltonian with the x2c-TZVPall basis set [37] and the x2c/J Coulomb-fitting auxiliary basis, with implicit aqueous solvation (SMD) [38], the DEFGRID3 numerical integration grid and TightSCF convergence. Thermochemistry was evaluated at the same level of theory at 298.15 K. All transition states were verified by IRC calculations connecting the intended precomplex and postcomplex minima [39].

Precomplex, transition-state and postcomplex structures were constructed with one coordinating water molecule hydrogen-bonded to the leaving group, the smallest explicit model that captures specific first-shell interactions. The entropy of association is treated analytically through the standard-state, solvent-concentration and Benson–Okuno corrections. Cysteine was modeled as the thiolate of acetyl-cysteinyl-N-methylamide (Ac-Cys-NHMe^−^), a truncated model of the glutathione thiol, and guanine as neutral 9-methylguanine. At pH 7.4, the thiol (p*K*_a_ = 8.80 [40]) is present as the thiolate to a fraction *f*_active_ = $\frac{1}{1+10^{pK_{a}-\mathrm{pH}}}$ = 3.83%, whereas 9-methylguanine (p*K*_a1_ = 3.3, p*K*_a2_ = 9.4 [41]) is predominantly neutral (*f*_active_ = $\frac{1}{1+10^{pK_{a_{1}}-\mathrm{pH}}+10^{\mathrm{pH}-pK_{a_{2}}}}$ ≈ 0.99).

Gibbs free energies were obtained from the energies of the isolated species and of the assemblies. Following a previously described protocol [42], three corrections were applied: the standard-state change (*SSS*, 1 atm → 1 M), the solvent-concentration change (*WSCA*, 1 M → 55.5 M) and the Benson–Okuno correction for the translational and rotational entropy lost when *n* molecules are combined into a supermolecule (*O-B*). The total correction is Δ*G*_corr_ = Δ*G_SSS_* + Δ*G_WSCA_* + Δ*G_O-B_*. For the present systems (*n* = 3: cisplatin, nucleophile and one water; Δ*n* = −2), Δ*G*_SSS_ = −3.79, Δ*G*_WSCA_ = −2.38, and Δ*G*_O-B_ = −4.92 kcal⋅mol^−1^, giving Δ*G*_corr_ = −11.09 kcal⋅mol^−1^.

The equilibrium constant for precomplex formation, *K*_pre_, follows from the free energy of the precomplex relative to the isolated reactants:

(1) $$K_{\mathrm{pre}}=\frac{\exp\left(-\Delta G_{\mathrm{pre}}^{{}^{\circ}}/\mathrm{RT}\right)}{c^{{}^{\circ}}},c{}^{\circ}=1\;M$$

Rate constants were obtained from conventional transition-state theory:

(2) $$k_{\mathrm{TST}}=\frac{k_{B}T}{h}\;\exp\left(-\frac{\Delta G^{‡}}{\mathrm{RT}}\right)$$

and the equivalent second-order rate constant is:

(3) *k_equiv_* = *K_pre_σk_TST_*

where *σ* is the symmetry number (2 for non-aquated, symmetrical cisplatin; 1 for all other species, including aquated cisplatin). Because *k*_equiv_ may approach or exceed the diffusion limit, the apparent rate constant was obtained from the Collins–Kimball equation [43]:

(4) $$k_{\mathrm{app}}=\frac{k_{\mathrm{equiv}}{k^{\prime }}_{D}}{k_{\mathrm{equiv}}+{k^{\prime }}_{D}}$$

with the diffusion-limited rate constant *k_D_* = 4*πR*_AB_*D*_AB_*N*_A_ × 10^3^ L⋅m^−3^ (*R*_AB_ in meters). The encounter distance *R*_AB_ is the sum of the effective radii of the reactants, and *D*_AB_ = *D*_A_ + *D*_B_, with the diffusion coefficients from the Stokes–Einstein equation (*η*_water_ = 0.8913 mPa⋅s at 298.15 K). For charged reactant pairs (mono-aquated cisplatin and thiolate), *k*_D_ was multiplied by the Debye factor *f* = *β*/(*e^β^*–1), so that *k*′_D_ = *fk_D_*, with *β* = *z*_Pt_*z*_Nu_*l*_B_/*R*_AB_, where *z*_Pt_ and *z*_Nu_ are the charges of the metal complex and the nucleophile, and *l*_B_ = 7.14 Å (Bjerrum length in water at 298.15 K); the factor is unity for neutral pairs. The observed rate constant for a nucleophile present at concentration [Nu] was:

(5) $$k_{\mathrm{obs}}=\frac{k_{\mathrm{app}}\left[\mathrm{Nu}\right]f_{\mathrm{active}}}{1+K_{\mathrm{pre}}\left[\mathrm{Nu}\right]f_{\mathrm{active}}}$$

with [Nu] × *f*_active_ = 3.83 × 10^−5^ M for the thiolate (1 mM total thiol), [Nu] × *f*_active_ = 9.90 × 10^−4^ M for guanine (1 mM total guanine, *f*_active_ ≈ 0.990), and [Nu] = 55.5 M for water. The relative preference of a complex for the thiolate over guanine is expressed as:

(6) $$S_{\mathrm{direct}}=\log_{10}\left(\frac{k_{\mathrm{app},\mathrm{Cys}}}{k_{\mathrm{app},\mathrm{Gua}}}\right)$$

The corresponding preference at the precomplex level is:

(7) $$S_{\mathrm{pre}}=\log_{10}\left(\frac{K_{\mathrm{pre},\mathrm{Cys}}}{K_{\mathrm{pre},\mathrm{Gua}}}\right)$$

The change in direct selectivity between the mono-aquated and non-aquated forms is defined as:

(8) Δ*S_mechanism_* = *S_direct_*_,_*_mono_*_−_*_aquated_* − *S_direct_*_,_*_non_*_−_*_aquated_*

As an independent reference for the magnitude of *K*_pre_, the association constant of a non-specific contact ion pair was estimated with the Fuoss equation [31], *K*_F_ = (4*πN*_A_*R*_AB_^3^/3)exp(−*β*), where β is defined as above and *R*_AB_ is expressed in dm, so that *K*_F_ is obtained in M^−1^. For mono-aquated cisplatin and thiolate, the value at an ionic strength of 0.1 M was also evaluated by dividing β by (1 + κ*R*_AB_), with κ = 0.329 *I*^1/2^ Å^−1^ (*I* in M). The stabilization of the calculated precomplex in excess of that of a contact pair is expressed as ΔΔ*G* = *RT* ln(*K*_pre_/*K*_F_). The Fuoss estimate assumes spherical ions interacting only electrostatically at contact distance; it is used here as an order-of-magnitude reference, not as a quantitative model for the anisotropic reactants.

An uncertainty of ±2–3 kcal⋅mol^−1^ is assumed for calculated barriers, which corresponds, through the Eyring equation at 298.15 K, to ±1.5–2.2 log units in an individual rate constant and ±2.1–3.1 log units (for independent errors in the two profiles) in *S*_direct_. Differences below these thresholds are not regarded as significant. The Collins–Kimball correction is only significant when *k*_equiv_ is comparable to *k*_D_; otherwise, *k*_app_ ≈ *k*_equiv_.

## 4. Conclusions

The protocol specifies every correction explicitly, so each can be tested independently. The calculated precomplex-to-transition-state barrier for aquation is 2.0 kcal⋅mol^−1^ below the Eyring barrier inferred from experiment; after applying the symmetry factor, the calculated observed rate constant is 56-fold higher than the experimental value. The formal low-concentration second-order coefficient for thiolation exceeds the reported glutathione rate coefficient by 1.5–1.8 log units, but this comparison does not constitute a concentration-matched prediction of the observed rate under conditions where the calculated precomplex is saturated. Because the aquation rate is insensitive to the encounter-complex free energy, this benchmark validates the barrier but not the term governing the thiolation rate and the thiolate/guanine ratio. To match the reported glutathione second-order rate coefficient in the formal low-concentration comparison, the thiolate precomplex would need to be destabilized by 2.1–2.5 kcal⋅mol^−1^; even then, it would remain 4.9–5.4 kcal⋅mol^−1^ above a Fuoss contact-pair estimate, indicating that electrostatic interactions alone cannot account for its stabilization. This stabilization may arise from genuine specific interactions or overstabilization by the implicit solvent, which the present data cannot disentangle. The sign of the thiolate/guanine preference is robust to errors of this magnitude, whereas its magnitude is not; consequently, only the direction of the preference can be reported confidently from such calculations.

The following steps are therefore proposed as a protocol for computing the substitution kinetics of Pt(II) complexes: (1) conformer sampling using GFN2-xTB/ALPB (GOAT); (2) optimization of stationary points at M06-2X/x2c-TZVPall with SMD solvation; (3) application of standard-state, solvent-concentration, and Benson–Okuno corrections; (4) computation of rate constants via transition-state theory with the Collins–Kimball diffusion limit; and (5) reporting of the four diagnostics to qualify the results. Without these corrections, the computed rates can deviate from experiment by several log units. Agreement between a computed barrier and experiment, when the calculation omits these corrections or employs an adjusted number of explicit water molecules, should not be considered evidence that the calculation is correct.

## Figures and Tables

**Table 1:** Calculated parameters for the reactions of cisplatin (298.15 K; energies in kcal⋅mol^−1^).

Pathway	Δ*G_(_*_pre−isol)_	*K*_pre_ (M^−1^)	Δ*G*^‡^_(pre–TS)_	Δ*G*_(TS–isol)_	Δ*G*_(post–isol)_	*k*_app_ (M^−1^ s^−1^)
Aquation	–1.5	1.2 × 10^1^	22.0	20.5	5.3	1.09 × 10^−2^
Thiolation, non-aquated	–7.3	2.4 × 10^5^	23.3	16.0	–21.3	2.28 × 10^1^
Guanine substitution, non-aquated	–1.3	9.2 × 10^0^	22.3	21.0	–4.8	4.86 × 10^−3^
Thiolation, mono-aquated	–8.3	1.3 × 10^6^	12.6	4.3	–32.1	3.13 × 10^9^
Guanine substitution, mono-aquated	1.0	1.8 × 10^−1^	12.8	13.8	–13.5	4.48 × 10^2^

*k*_app_ for the thiolate pathways is defined with respect to the free thiolate concentration and is multiplied by *f*_active_ when compared with experimental coefficients based on total thiol. For the aquation pathway *k*_app_ = *K*_pre_ × *k*_obs_, and the quantity compared with experiment is *k*_obs_ = 9.06 × 10^−4^ s^−1^. *S*_direct_ = 3.7 (non-aquated) and 6.8 (mono-aquated).

**Table 2:** Effect of protocol settings on calculated quantities for cisplatin (one change at a time).

Change	log(*k*_obs,aq_/*k*_exp_)	log(*k*_Cys_/*k*_exp_)	*S*_direct_ (non-aq.)	*S*_direct_ (mono-aq.)
Baseline protocol	1.75	1.51	3.67	6.84
*Physical corrections*				
No standard-state correction (1 atm → 1 M)	1.47	−1.27	3.67	6.98
No solvent-concentration correction (1 M → 55.5 M)	1.71	−0.23	3.67	6.97
No Benson–Okuno correction	0.90	−2.10	3.67	6.98
None of the three corrections	−3.56	−6.62	3.67	6.98
Barrier from Δ*E*_el+ZPE_ instead of Δ*G*	2.74	1.33	3.62	6.14
No Collins–Kimball correction	1.75	1.51	3.67	6.98
No Debye (electrostatic) factor	1.75	1.51	3.67	6.78
Symmetry factor σ: 2 → 1 (non-aquated cisplatin)	1.45	1.21	3.67	6.84
*Sensitivity tests*				
*K*_pre_ capped at 10^2^ M^−1^	1.75	−1.86	0.30	2.88
*K*_pre_ capped at 10^4^ M^−1^	1.75	0.14	2.30	4.88
All barriers +2 kcal⋅mol^−1^	0.28	0.04	3.67	6.97
All barriers −2 kcal⋅mol^−1^	3.21	2.98	3.67	5.92
All precomplexes +2 kcal⋅mol^−1^	1.73	0.04	3.67	6.97
All precomplexes −2 kcal⋅mol^−1^	1.75	2.98	3.67	5.92
*Experimental parameters*				
Thiol: pH 7.0, pK_a_ 8.8 (*f*_active_ = 1.6%)	1.75	1.12	3.67	6.84
Thiol: pH 7.8, pK_a_ 8.8 (*f*_active_ = 9.1%)	1.75	1.89	3.67	6.84
Thiol: pH 7.4, pK_a_ 8.5 (*f*_active_ = 7.4%)	1.75	1.79	3.67	6.84
Thiol: pH 7.4, pK_a_ 9.2 (*f*_active_ = 1.6%)	1.75	1.12	3.67	6.84

*k*_exp_ = 1.62 × 10^−5^ s^−1^ (aquation, 298 K) [17] and 2.7 × 10^−2^ M^−1^ s^−1^ (glutathione, 310 K) [21]; the alternative experimental value, 1.3 × 10^−2^ M^−1^ s^−1^ [22], would shift all log(*k*_Cys_/*k*_exp_) entries by +0.32, corresponding to log(2.7/1.3). *k*_Cys_ = *k*_app,Cys_ × *f*_active_ for the non-aquated pathway; [Nu] = 1 mM, so [Nu] × *f*_active_ = 3.83 × 10^−5^ M. *S*_direct_ (non-aq.) and *S*_direct_ (mono-aq.) refer to non-aquated and mono-aquated cisplatin, respectively.

**Table 3:** Comparison of the calculated precomplex constants with the Fuoss estimate for a non-specific contact ion pair (298.15 K).

Pathway	*R*_AB_ (Å)	*z*_Pt_ *z*_Nu_	*K*_F_ (M^−1^)	*K*_pre_ (M^−1^)	log (*K*_pre_/*K*_F_)	ΔΔ*G* (kcal⋅mol^−1^)
Thiolate + cisplatin	6.99	0	0.86	2.4 × 10^5^	5.44	7.4
Guanine + cisplatin	6.74	0	0.77	9.2	1.08	1.5
Thiolate + mono-aquated cisplatin	6.79	−1	2.26	1.3 × 10^6^	5.74	7.8
Guanine + mono-aquated cisplatin	6.54	0	0.71	0.18	−0.59	−0.8

*K*_F_ = (4*πN*_A_*R*_AB_^3^/3)exp(−*z*_Pt_*z*_Nu_*l*_B_/*R*_AB_) [31], where *l*_B_ = 7.14 Å is the Bjerrum length at zero ionic strength. At I = 0.1 M, *K*_F_ of thiolate with mono-aquated cisplatin is 1.5 M^−1^. ΔΔ*G* = *RT* ln(*K*_pre_/*K*_F_).

## Data Availability

The raw data supporting the conclusions of this article will be made available by the authors upon reasonable request.
